# Assisted reproduction after SARS-CoV-2-infection: results of a single-center cohort-study

**DOI:** 10.1007/s00404-023-07228-w

**Published:** 2023-10-10

**Authors:** Violet Eckstein, Katrin Glaß, Marie-Elisabeth Leßmann, Jessica Schaar, Anna Klimova, Pauline Wimberger, Maren Goeckenjan

**Affiliations:** 1 Department of Gynecology and Obstetrics, University Hospital Carl-Gustav-Carus, Technische Universität Dresden, Fetscherstraße 74, 01307 Dresden, Germany; 2 Institute for Medical Informatics and Biometry, Technische Universität Dresden, Fetscherstraße 74, Dresden, Germany

**Keywords:** COVID-19, Omicron, Pregnancy, Miscarriage, IVF

## Abstract

**Purpose:**

The effects of SARS-CoV-2 infections on the outcome of assisted reproduction techniques (ART) were studied in a retrospective cohort study.

**Methods:**

The outcome of 1581 treatment cycles with embryo transfer at a university fertility center in Germany was compared in years before and during the COVID-19 pandemic. For 335 treatment cycles in 2022 a detailed analysis was carried out depending on infection and immunization status of both partners.

**Results:**

ART cycles did not differ in most of the parameters examined between 2018–2022. In spite of comparable clinical pregnancy rates, there was a significantly higher miscarriage rate at 34.6% (27/78) in 2022, compared to 19.7% (29/147) in the pre-pandemic years of 2018–2019 (*p* = 0.014). In 37.0% of the treatment cycles (124/335) 2022 at least one partner reported a SARS-CoV-2-Infection 6 months before ART, mostly with the virus variant Omicron. Clinical pregnancy rates were lower in cycles without infection. Comparing women with confirmed infection to no infection, a significantly higher risk of miscarriage was seen (62.5% vs. 26.2%, *p* = 0.009). In treatment cycles of partners with basic immunization against SARS-CoV-2 a statistically significant increase of pregnancy rates was seen comparing to cycles with both unvaccinated partners (*p* = 0.011).

**Conclusion:**

The results indicate a negative impact of SARS-CoV-2-infections up to 6 months on ART treatment, in particular an increased risk of miscarriage. Vaccination was associated with a better outcome of ART treatment.

**Supplementary Information:**

The online version contains supplementary material available at 10.1007/s00404-023-07228-w.

## What does this study add to the clinical work

**Table:** 

This study shows an impaired outcome after assisted reproduction in 2022 compared to the years before the pandemic in a single center. A significantly lower clinical pregnancy rate was found in ART cycles after SARS-CoV-2 infection in the 6-month period before treatment compared with couples without confirmed infection, additionally a significantly higher risk for miscarriages was found in women with SARS-CoV-2 infections.	

## Introduction

**Fig. 1:**
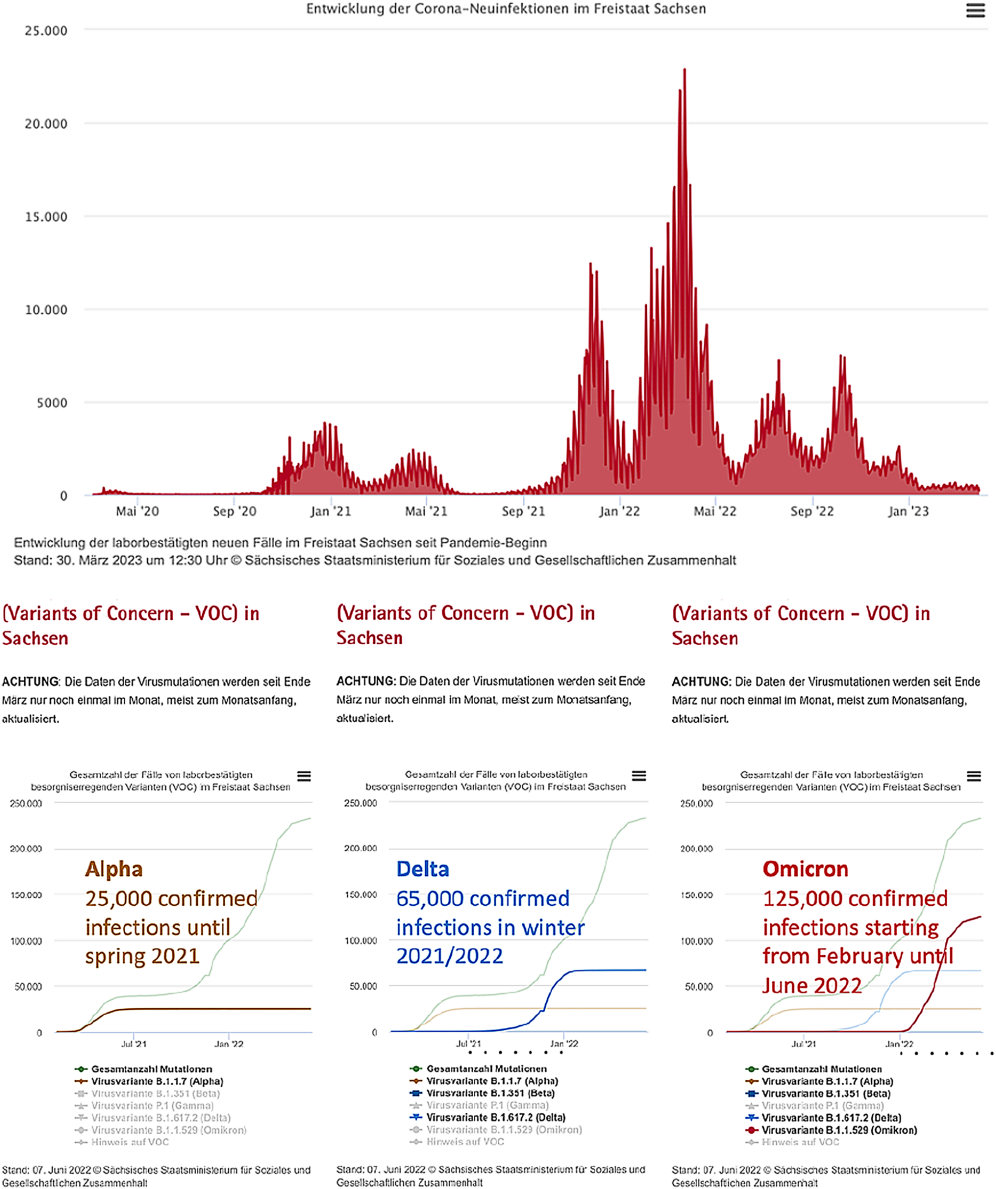
Confirmed SARS-CoV-2-infections in Saxony (http://www.coronavirus.sachsen.de, https://www.coronavirus.sachsen.de/infektionsfaelle-in-sachsen-4151.html#a-8991, accessed 12 May 2023) and dominant virus variants (http://www.coronavirus.sachsen.de, accessed 7 June 2022, no longer available in May 2023)

During the COVID-19 pandemic the highest number of confirmed infections in the population in Germany occurred in 2022. Regional and national news published daily updated numbers of confirmed infections with SARS-CoV-2. In total, almost 2 million confirmed coronavirus infections were registered in Saxony by May 2023 in a population of 4 million inhabitants.^1^ At the same time, 68.8% of the population of Saxony aged 18–59 years had been vaccinated at least once since December 2020, 67.5% received basic immunization with 2 vaccinations, a first booster was given in 44.3% and a second booster in 3.4%.^2^ The wave of SARS-CoV-2-infections with the strongest impact on public life, such as restrictions on daily life, intensive care treatment of infected persons and the highest relatively virus-associated death rate, occurred in Saxony in winter of 2020/2021, before vaccinations were established. However, most infections occurred from winter 2021/2022 onwards (Fig. 1).

Based on official data publications, it was possible to determine which waves of infection were significantly caused by which virus variants (Fig. 1). For the year 2022 this was mainly the virus variant Omicron and to a lesser extent the variant Delta. During the COVID-19 pandemic treatments with assisted reproductive techniques (ART) with in vitro fertilization (IVF) or intracytoplasmic sperm injection (ICSI) were performed at a University Hospital in Saxony in comparable numbers as in previous years, except for 6 weeks during spring 2020. First couples showed confirmed SARS-CoV-2 infections before or during treatment in winter 2021/2022.

In the early phases of the pandemic, international guidelines advised against performing fertility treatment except in urgent cases, due to infection control and the potential impact of SARS-CoV-2 infections on pregnancy outcomes [1]. In September 2020, three international societies of reproductive medicine released a consensus statement, acknowledging the need for assisted reproduction [1]. After careful evaluation of local conditions and well-being of patients, along with adherence to governmental regulations to reduce the risk of viral transmission and counseling of the infertile couples, ART was recommended. Additionally, research on the potential impact of SARS-CoV-2 on reproduction was encouraged.

Current studies have not shown consistent results concerning the effects of SARS-CoV-2 infections on the outcome of fertility treatment or early pregnancy. However, a negative impact of SARS-CoV-2 infections on parameters of sperm quality up to 3 months after infection has been demonstrated [2]. This was also confirmed in a prospective study of 120 men after SARS-CoV-2 infections [3]; fever and severity of symptoms had no impact on sperm quality. The German registry CRONOS prospectively collects data on SARS-CoV-2 infections in pregnancy [4]. In this registry, infections in early pregnancy can also be reported, yet a possible reporting bias with predominance of later and severe infections in pregnancy should be considered.

In spring 2022, the University Fertility Center noticed an unexpectedly high number of pregnancies after ART ending in miscarriages. In order to clarify the question whether SARS-CoV-2 infections in the context of assisted reproduction could lead to an increase in miscarriage rate, an observational and questionnaire-based study was conducted. The infection and vaccination status of treated couples were also analyzed.

## Methods

Between 01/2018 and 12/2022, in total 1716 ART cycles were performed at the university Hospital. Study-relevant clinical data were extracted from the clinical documentation system and the MediTEX program used for ART treatments. Data on SARS-CoV-2 infections and immunizations as well as the follow-up in pregnancy were collected by telephone interview conducted by 3 researchers (VE, MG, JS). The study protocol was approved by the local ethics committee (BO-EK-349082022).

**Fig. 2:**
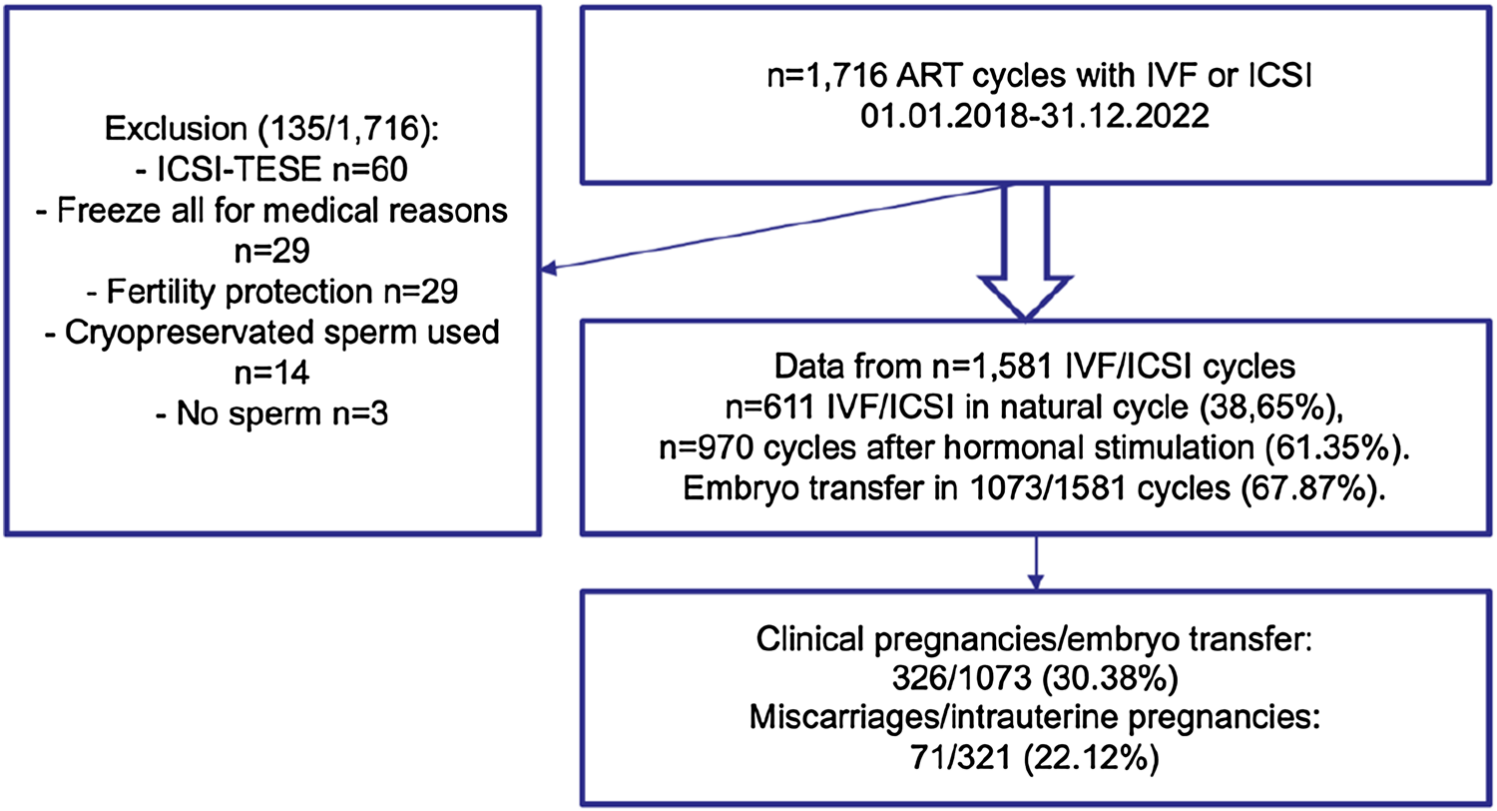
Flow diagram of study inclusion (treatment cycles over 5 years), 5 of 326 pregnancies were excluded for calculation of miscarriage rate because of ectopic location

A total of 1581 treatment cycles with stimulated or natural cycle and planned embryo transfer without gamete donation were included in the study (Fig. 2). Exclusion criteria were use of cryopreserved sperm, oocytes, and embryos and ART cycles with freeze-all after oocyte retrieval.

For further information see Supplementary Information. Two couples of 214 couples treated with ART in 2022 were not interviewed due to language difficulties. One couple was not reached. The response rate in the questionnaire study was 98.6%. The last follow-up in pregnancy was at 22 gestational weeks. The birth rate could not be calculated in May 2023 yet. The telephone interview using a structured questionnaire (see Supplementary Information) was conducted on average 3.3 ± 1.2 (0.5–5.2) months after ART and was repeated 3 months later during pregnancy. A SARS-CoV-2 infection was considered as confirmed by positive polymerase chain reaction (PCR) test from nasopharyngeal swab.

### Statistics

Statistical analysis was performed using IBM SPSS version 25 program (SPSS Inc., Chicago, IL, USA). Categorical data are presented as absolute and relative frequencies, metric data are presented as mean, standard deviation, minimum, and maximum, or alternatively as median with 25th and 75th percentiles. Group comparisons for categorical variables were compared using Pearson's Chi-square tests. The Shapiro–Wilk test was used to test for normal distribution of metric data. Non-normally distributed data were analyzed using the Mann–Whitney-*U* or Kruskal–Wallis test. In all statistical tests, a *p*-value < 0.05 was considered as statistically significant.

## Results

### ART treatments before and during the COVID-19 pandemic

A total of 1581 ART cycles from 2018 to 2022 were included for comparison. In 38.6% of cycles, oocyte retrieval was performed in the natural cycle (611/1581). Women were primarily infertile in 56.6% (895/1580) of cycles, and IVF was performed in 45.5% (719/1581). The mean age of patients was 35.8 ± 4.1 (23.1–49.5) years. There were no significant differences in the number of cycles per year despite limited treatment options in 2020 during the beginning of the pandemic. Regarding the data from 2018 to 2022, the average clinical pregnancy rate per embryo transfer was 30.4% (326/1073) and the miscarriage rate was 22.1% (71/321 intrauterine pregnancies) after ART. Comparing the yearly outcome parameter, there were significant differences in the number of embryos per transfer (*p* < 0.001) due to changes in standards at the center with the introduction of prolonged embryo culture in 2019. However, no significant difference was found considering the average clinical pregnancy rate (*p* = 0.113). Clinical pregnancy rates ranged from 25.0–35.5%/year. In 2018–2021, the annual miscarriage rate was 11.1–25.0% compared to 34.6% in 2022 (*p* = 0.014).

Table 1 shows the course of 970 ART cycles after controlled ovarian stimulation. For further detailed analysis, the years 2018 and 2019 before the onset of the pandemic were compared to 2022, when SARS-CoV-2 infections frequently occurred. The patients were 1 year older in 2022, with a mean age of 35.7 ± 4.0 years vs. 34.9 ± 4.2 years in 2018–2019 (*p* < 0.001). Significant differences were also observed when comparing 2018/2019 to 2022 concerning primary infertility, fertilization rates and number of oocytes with 2 pronuclei (2PN). Nevertheless, comparably high pregnancy rates of 38.2% in 2018/2019 and 38.5% in 2022 were found (*p* = 1.000). However, a statistically significant difference was found regarding the miscarriage rate of 20.5% before the pandemic vs. 37.1% in 2022 (*p* = 0.015). The miscarriages occurred at 8.0 ± 2.5 (6–18) weeks of gestation, the age of women at miscarriage was 35.4 ± 4.2 (23.4–43.8) years. Half of the women were pregnant for the first time, and 6/27 (22.2%) had a previous miscarriage.

**Table 1:** Treatments with IVF/ICSI after controlled ovarian stimulation (*n* = 970)

	2018 and 2019 (*n* = 403)	2022 (*n* = 189)	*p*-value (Chi-quadrat-test or Mann–Whitney *U*)	All 5 years (*n* = 970)
Age (years)*	34.85 ± 4.17 (25.51–49.46) 35.72 (32.73; 39.11)	35.68 ± 4.01 (23.36–44.28) 36.16 (32.96; 38.77)	*p* < 0.001	35.19 ± 3.77 (25.29–46.71) 35.31 (32.6; 38.18)
Primary infertility*	261/403 (64.67%)	102/189 (53.97%)	*p* = 0.014	562/969 (58.00%)
Cycles with conventional IVF	181/403 (44.91%)	91/189 (48.15%)	*p* = 0.480	449/970 (46.29%)
Embryo transfer in cycles with oocytes	246/391 (88.49%)	161/187 (86.10%)	*p* = 0.419	805/949 (84.83%)
Blastocyst culture, if > 2 fertilized oocytes*	64/227 (28.19%)	92/120 (76.67%)	*p* < 0.001	320/565 (56.64%)
Number of oocytes/retrieval	8.91 ± − 5.43 (0–36) 8 (5; 12)	8.99 ± 5.10 (0–24) 8 (5; 12)	*p* = 0.742	8.83 ± 535 (0–36), 8 (5; 12)
Number of fertilized oocytes*	3.60 ± 2.70 (0–15) 3 (2; 5)	4.15 ± 2.80 (0–13), 4 (2; 6)	*p* = 0.019	3.66 ± 2.77 (0–17), 3 (2; 5)
Fertilized oocytes/retrieved oocytes*	42.32 ± 26.14 41.67 (25; 58.33)	47.98 ± 24.85 46.67 (33.3; 62.5)	*p* = 0.006	43.22 ± 25.58 42.86 (25; 60)
Number of embryos/transfer*	1.75 ± 0.51 (1–3) 2 (1; 2)	1.54 ± 0.51 (1–3) 2 (1; 2)	*p* < 0.001	1.60 ± 0.52 (1–3) 2 (1; 2)
Clinical pregnancy rate	132/346 (38.15%)	62/161 (38.50%)	*p* = 1.000	284/805 (35.28%)
Miscarriage/pregnancy*	27/132 (20.45%)	23/62 (37.10%)	*p* = 0.015	63/282 (22.26%)

*Significant group differences < 0.05

### SARS-CoV-2 infection associated with ART cycles

Analysis of 335 ART cycles in 2022 with available information on SARS-CoV-2 infection showed that in 51 cycles (15.2%) both partners had PCR-test-confirmed SARS-CoV-2 infection occurring in a window of 6 months before pregnancy test. In 73 cycles, one of the two partners (21.8%) had a confirmed infection, in 211 cycles, no infection was reported (63.0%). In the course of treatment, there were no statistically significant differences in treatment characteristics between couples with and without SARS-CoV-2 infection (Table 2). However, there was a significant difference in pregnancy rates of 21.8% in the group with infection compared to 38.8% without infection (*p* = 0.010). The miscarriages rates in couples with infection were 52.6% vs. 27.6% without infections, although this difference was not statistically significant (*p* = 0.055). When considering the 85 ART-cycles (25.4%) in which the woman had an infection less than 6 months prior to treatment, a significantly higher miscarriage rate was seen compared to cycles without infection (62.5% vs. 26.3%, *p* = 0.009). The clinical pregnancy rate was also lower in women with infection (22.2%) than in women without infection (35.8%), but not statistically significant (*p* = 0.092).

**Table 2:** Characteristics of ART-cycles in comparison of couples with/without SARS-CoV-2-infections (2022)

	All cycles (*n* = 335)	One or both partner with infections* (*n* = 124)	No infection^1^ (*n* = 211)	Mann–Whitney-*U* Chi-quadrat test (exact 2-sided)
Age (years)	35.73 ± 3.99 (23.36–44.28) 36.16 (33.08; 38.79)	35,96 ± 3.91 (23.36–42.87) 36.14 (34.04; 39.10)	35.59 ± 4.05 (23.79–44.28) 36.29 (32.77; 38.68)	*p* = 0.411
Primary infertility	178/335 (53.13%)	61/124 (49.19%)	117/211 (55.45%)	*p* = 0.308
IVF treatment	176/335 (52.54%)	58/124 (46.77%)	118/211 (55.92%)	*p* = 0.114
Natural cycle IVF	149/335 (44.48%)	64/124 (51.61%)	89/211 (42.18%)	*p* = 0.306
Embryo transfer in cycles with oocytes	239/306 (78.10%)	87/114 (76.32%)	152/192 (79.17%)	*p* = 0.570
Blastocyst culture, if > 2 fertilized oocytes	93/123 (75.61%)	31/42 (73.81%)	62/81 (76.54%)	*p* = 0.826
Number of oocytes/retrieval	5.50 ± 5.52 (0–24) 3 (1; 9)	5.17 ± 5.41 (0–24), 2 (1; 9)	5.69 ± 5.59 (0–23) 4 (1; 9)	*p* = 0.454
Number of fertilized oocytes	2.84 ± 2.79 (0–13) 2 (1; 4)	2.69 ± 2.83 (0–12), 1.5 (1; 4)	2.92 ± 2.00 (0–13) 2 (1; 4)	*p* = 0.293
Fertilized oocytes/ retrieved oocytes	52.87 ± 35.12 (0–100) 50 (2.90; 93)	53.23 ± 36.17 50 (26.70; 100)	52.66 ± 34.58 (0–100) 50 (28.93; 90)	*p* = 0.925
Number of embryos/transfer	1.42 ± 0.52 (1–3) 1 (1; 2)	1.37 ± 0.51 (1–3) 1 (1; 2)	1.45 ± 0.53 (1–3) 1 (1; 2)	*p* = 0.243
Clinical pregnancy rate*	78/239 (32.64%)	19/87 (21.84%)	59/152 (38.82%)	*p* = 0.010
Miscarriage/pregnancy	26/77 (33.77%)	10/19 (52.63%)	16/58 (27.59%)	*p* = 0.055

*Significant group differences < 0.05

^1^At least 1 partner with a proven SARS-CoV-2-infection within < 6 months prior to oocyte retrieval/pregnancy test

Possible influences of vaccination against SARS-CoV-2 on the course of ART cycles were also investigated in the study. In 228 of 335 ART cycles, both partners had basic immunization (68.1%). Basic immunization of only one partner was present in 39 cycles (11.6%). 68 cycles were in couples without vaccination (20.3%). There was a significantly higher pregnancy rate in cycles with both vaccinated partners (57/168, 33.4%) and one partner (13/23, 56.5%) compared with non-vaccinated couples (10/48, 20.8%) (*p* = 0.011). The miscarriage rate did not differ among the three groups (*p* = 0.751).

## Discussion

In 2022, a large proportion of the German population was affected by SARS-CoV-2 infections for the first time. This retrospective study at a university fertility center shows that compared to previous years 2018–2021, miscarriages were significantly more frequent in 2022 (*p* = 0.014). SARS-CoV-2 infections less than 6 months before ART in at least one partner had a negative impact on pregnancy rate and outcome.

### ART treatments during the COVID-19 pandemic

In Germany, the number of fertility treatments did not decrease during the COVID-19 pandemic [5]. Couples may have initially postponed infertility treatments in 2020. Due to the pandemic and unpredictable risks, many fertility centers, especially university-affiliated centers, had to reduce elective procedures to a minimum of care in spring 2020. Nevertheless, the number of ART treatments has not changed significantly over the last 5 years. Couples may have been particularly focused on the issue of starting a family during lockdown. Although the pandemic caused anxiety and concerns about fertility and family formation, the overall desire to have children remained strong [6]. A particularly high psychosocial burden of the COVID-19 pandemic on infertile couples has been described for severely affected regions such as Italy [7].

The couples in the study were vaccinated with comparable frequency to the general population in Saxony.

The mean age of the treated women in the study was 35.8 years, which is in line with the current mean age of the German IVF Registry (DIR) data (see Table 3, [8–11]). The characteristics of the ART treatments reported in this study were also comparable with the 2018–2021 DIR data in terms of pregnancy and miscarriage rates. However, the miscarriage rate observed in 2022 of 37.1% is significantly higher. Outcomes of ART treatments in Germany for 2022 are not yet available.

**Table 3:** Pregnancy, miscarriage and birth rates after ART in Germany in the last 5 years as reported by the German IVF registry

Year	Age of women (years)	Number of ART-treatment with fertilisation	Clinical pregnancy rate in “fresh cycles”	Miscarriage rate (per pregnancy)	Birth rate (per embryo transfer)	Time of publishing
2018	35.2	65.328	32.2%	23.6%	19.8%	Jahresbericht 2018, June 2019 (8)
2019	35.5	61.188	32.7%	23.3%	20.4%	Jahresbericht 2019, May 2020 (9)
2020	35.6	66.447	32.7%	21.6%	23.5%	Jahresbericht 2020, May 2021 (10)
2021	35.7	71.602	31.8%	not published yet	not published yet	Jahresbericht 2021, May 2022 (11)
2022	–	not published yet	not published yet	not published yet	not published yet	

### Risks of SARS-CoV-2 infection in early pregnancy

An increased risk of miscarriage has been discussed for viral infections [12]. These include systemic infections such as cytomegalovirus, hepatitis C virus, and influenza virus. During the early stage of the pandemic, it was suspected that SARS-CoV-2 infections might lead to an increased risk of miscarriage. A systematic review considered 11 case reports and series that indicated an increased risk of miscarriage most likely due to trophoblastic or placental inflammation [13]. Vascular changes such as activation of coagulation and vasculitis in the uteroplacental stromal bed induced by SARS-CoV-2 infection may contribute to miscarriages [14].

Few SARS-CoV-2 infections occurred in young women during the first months of the pandemic. This was also shown in a study of 1019 pregnant women in Denmark in the spring of 2020 [15]. Only 1.8% of women had serological evidence of having passed through infection without increased risk of severe infection or high-risk pregnancy outcomes. A UK online survey study of the risk of miscarriage following SARS-CoV-2 infection in the first year of the pandemic found an increased risk of miscarriage [16]. Based on data from 3041 women, miscarriages occurred at an average of 9 weeks' gestation. The relative risk was 1.7 in the group of women with infection compared to women without infection (95% confidence interval 1.0–3.0, *p* = 0.06).

Studies of pregnancy outcomes in SARS-CoV-2 infection have mostly examined infections during pregnancy, while pre-pregnancy and early pregnancy infections have not been considered [17]. The prospective registry study from Germany CRONOS is based on reports of pregnant women with SARS-CoV-2 infections (3). 20.5% of the 3481 pregnant women had documented SARS-CoV-2 infections in the first trimester before 14 + 0 weeks of gestation. Based on data from 17 miscarriages, the risk of miscarriage was estimated to be less than 2% by 4 weeks after first detection of infection in the first trimester [3].

A recent study used national monthly incidence rates of spontaneous miscarriage between 2017–2021 in Israel to examine potential effects of the COVID-19 pandemic [18]. The average observed miscarriage rate was 22.0% with a slight upward trend. A calculated prediction for the third wave with the Omicron virus variant could range from 22.8 to 25.3%.

### SARS-CoV-2 infections and ART treatments

Studies on the impact of SARS-CoV-2 infection on ART treatment course have presented conflicting results. For example, a cohort study from Italy found no significant differences in implantation rates, clinical pregnancy rates, and miscarriage rates for the first year of the pandemic with 749 treatment cycles compared with 844 treatments that had occurred in 2019 [19]. Only women without evidence of active SARS-CoV-2 infection were treated: “COVID-free protocol” to protect patients and treatment providers. Another cohort study from Italy with data from ART treatments during the dramatic pandemic situation in asymptomatic patients showed no difference in treatment outcome compared with 2019 [20]. From CRONOS trial data, 65 pregnancies after ART were compared with 1420 spontaneously conceived pregnancies [21]. Severe courses of SARS-CoV-2 infection were not more common in women with ART pregnancies than in the comparison group. An increased risk of pregnancy complications after assisted reproduction was due to several factors such as older age, multiple pregnancies, and BMI over 30. The risk of miscarriage could not be studied due to insufficient numbers.

### Effects of SARS-CoV-2 vaccination on ART outcome and miscarriage rates

Recent Studies tend not to show an increased risk for an unfavorable course of fertility treatment or early pregnancy due to SARS-CoV-2 vaccination. For example, a systematic meta-analysis shows no adverse effects of vaccination against SARS-CoV-2 on fertility, infertility, or the course of fertility treatment [22]. Another more recent meta-analysis of 20 studies with a total of 18,877 ART treatment cycles also showed no significant negative effects of vaccination against SARS-CoV-2 on treatment outcome compared with nonvaccinated women [23]. A population-based study of 18,780 women with miscarriages from the United Kingdom found no association of vaccination with miscarriages compared with unvaccinated women before the pandemic (adjusted odds ratio of 1.02, 95% CI of 0.96–1.09 [24]). A Norwegian study showed a lower relative risk of miscarriage in women after vaccination [25]. A Spanish retrospective observational study of 510 women with SARS-CoV-2 vaccination found no adverse effects on ART treatment course and ovarian reserve comparing the different vaccines [26]. This study did not have a comparison group of non-vaccinated women or a comparison group of women with and without proven infections.

In our study, SARS-CoV-2 vaccination was also not associated with negative effects on the course of ART treatment—in fact, in treatment cycles where partners had basic immunization against SARS-CoV-2, statistically significantly higher pregnancy rates were found than in cycles with both unvaccinated partners (*p* = 0.011). It is likely that the effect is not due to vaccination directly but to risk reduction of infection or severe infection [22].

### Strengths and limitations

During spring 2022, a higher number of miscarriages after ART than expected occurred at our center. We planned a retrospective, monocentric cohort analysis with prospectively collected data from summer 2022 onwards. Data from 211 couples on SARS-CoV-2-infections and vaccinations were collected using a structured short questionnaire. At the time of the study, self-testing was routine, SARS-CoV-2 infections were confirmed in a standardized manner by PCR testing, and the impact of SARS infection on daily life was severe in 2022. Therefore, the self-reported information of t time and symptoms can be considered reliable. Other strengths of the study include the high inclusion of 335/338 possible couples with 99.1% participation rate in the survey. Infection and vaccination status of both partners were assessed. The rapid analysis and publication of data, even before data from the IVF registry are available, is also a strength of the study. There are no comparable studies of miscarriage rates after spontaneous conception. Women with early miscarriages are increasingly treated conservatively with a wait-and-see strategy without the need for curettage [27]. A change in the number of miscarriages in Germany is difficult to estimate.

The data on infections were based on self-reporting of the study participants. Further limitations of the study are the possible recall and interviewer bias. A reliable antibody status for SARS-CoV-2 infections could not be used for the study, as a clinical marker for SARS-CoV2-immunity has not been established at the time of the study [28]. Asymptomatic infections may be underreported, and the proportion of undetected infections can be estimated 1.5–4 times higher than the number of confirmed cases [28]. Regarding infections < 3 months before ART treatment, the small numbers in this study in each group limited the statistical analysis. Nevertheless, the effect on miscarriage rate was also high after infection in a time-frame of 3 months prior to ART (50.0% (5/10) after infection vs. 30.9% (21/68) without infection, *p* = 0.292).

## Conclusion

This monocentric study indicates that SARS-CoV-2 infections in couples prior to ART treatment may result in a lower pregnancy rate. In particular, infections in the woman were associated with a higher risk of miscarriage in this study. It is not yet clear whether these risks will also be confirmed in the German IVF Registry. Possible consequences for further phases with high numbers of SARS-CoV-2 infections and future virus variants would be that couples wishing to have children should wait at least 3–6 months after an infection before starting ART treatment.

### Supplementary Information

Below is the link to the electronic supplementary material.

Supplementary file1 (DOCX 1141 KB)

## Data Availability

The data that support the findings of this study are available on request from the corresponding author MG upon reasonable request. The data are not publicly available due to protection of privacy of included patient data.

## Footnotes

1 https://www.coronavirus.sachsen.de/infektionsfaelle-in-sachsen-4151.html#a-8998, accessed 12 May 2023.

2 https://www.coronavirus.sachsen.de/ueberblick-coronaschutzimpfungen-in-sachsen-9874.html, accessed 26 May 2023.
