# Supplementary material for: Assisted reproduction after SARS-CoV-2-infection: results of a single-center cohort-study
Source: Arch Gynecol Obstet. 2023 Oct 10;309(1):305–13. doi: 10.1007/s00404-023-07228-w (PMC10769908; doi:10.1007/s00404-023-07228-w)

**Supplementary Information**

**Outcome of treatments with assisted reproductive technology after SARS-CoV-2-infection**

**Violet Eckstein^1^, Katrin Glaß^1^, Marie Lessmann^1^, Jessica Schaar^1^, Anna Klimova^2^, Pauline Wimberger^1^, Maren Goeckenjan^1^**

1. Department of Gynecology and Obstetrics, University Hospital Carl-Gustav-Carus, Technische Universität Dresden, Germany
2. Institute for medical biometrics and biometry, Faculty of Medicine, Technische Universität Dresden, Germany

**Description of ART treatments with IVF/ICSI**

The following "Corona" regulations applied for the entire year 2022 in the outpatient clinic of the University Women's Hospital: In addition to the FFP2 mask obligation in the entire clinic area for patients and employees, the "3G status" was checked upon entering the clinic. All patients received a wristband with a code. Unvaccinated patients had to present a daily antigen test.

In 2022, a total of 211 couples were treated. Couples had an average of 2.1±1.7 (1-11) treatments in the natural cycle and 1.4±0.8 (1-6) treatments in the stimulated cycle. 75 couples were treated 2x, 31 couples 3x. 19 couples received more than 3 treatments during the study period, including one patient who had 11 oocyte retrievals in the natural cycle without pregnancy. In her case, after ovarian transplantation, every natural cycle was used for ART if possible.

611 IVF/ICSI ART-cycles were performed in natural cycles with or without stimulation with clomiphene (25 mg for 5 days) or letrozole (2.5 mg for 5 days). Final oocyte maturation was induced after sonographic visualization of a preovulatory follicle of at least 16 mm with recombinant human chorionic gonadotropin (250 µg). Upon detection of a rise in luteinizing hormone (LH) above 7.5 U/l, the patient was advised to take 3x50 mg of diclofenac orally until follicular puncture. Oocyte retrieval was performed ultrasound-guided 35 h after ovulation triggering, mostly without further analgesics or anesthesia.

In 970 ART-cycles, oocyte retrieval took place after hormonal stimulation. All patients were treated in standardized stimulation protocols. For the majority of patients, the long gonadotropin-releasing hormone agonist protocol was used, and less frequently the antagonist protocol or the modified low-stimulation protocol. Mostly FSH was used for stimulation, less frequently the combination of recombinant FSH with LH or human menopausal gonadotropin in doses between 100 and 225 IU/die. Transvaginal ultrasound with determination of follicle number and size as well as endometrial thickness were performed regularly until dominant follicles had a follicle size of 18 mm. In cycles after controlled ovarian stimulation (KOS), follicular puncture took place 35 h after ovulation triggering with 250 µg recombinant hCG mainly under anesthesia with administration of propofol and fentanyl.

After ejaculation at the time of oocyte retrieval, sperm processing was performed by swim-up method. About 4 h, 2-3 oocytes each were incubated in a well containing 1 ml culture medium (G-IVF, Vitrolife) and about 100,000 progressively motile sperm per oocyte for IVF. For ICSI treatment, the oocyte was denuded with Hylase 1ml/well approximately 3 h after follicular puncture and the cumulus complex was mechanically removed. Using a micromanipulator with 200x magnification, spermatozoa were mechanically immobilized and aspirated into a injection needle. After fixation of the oocyte with a holding pipette, the sperm was injected into the cytoplasm. The control for fertilization took place the next morning, approximately 21 h after follicular puncture.

Depending on the number of fertilized oocytes and after informed consent, embryo transfer was performed. On day 2 to 3 1 or 2 embryos were transferred, in rare cases 3 embryos. Planned single embryo transfer was mostly performed after prolonged embryo culture on day 5. 3x10 mg dydrogesterone were mainly used as luteal support. In case of positive detection of hCG in serum 12 days after blastocyst transfer, or 14 days after transfer on day 2-3 after oocyte retrieval, this substitution was continued until 10 weeks of gestation.

**Definitions of outcome variables:**

**Assisted reproductive technology (ART):** Treatments with oocyte retrieval after hormonal stimulation or in natural cycle and subsequent fertilization of the egg with IVF/ICSI.

**Miscarriage:** termination of pregnancy after evidence of an intrauterine amniotic cavity before 22 SSW

**Fertilization rate:** Number of oocytes with 2 pronuclei (2PN) / number of oocytes retrieved in %. For comparability of all cycles, differentiation between IVF and ICSI methods was omitted.

**Clinical pregnancy rate:** Number of treatments with evidence of an amniotic cavity / number of embryo transfers in the studied groups in %.

**Miscarriage rate:** Number of pregnancies with miscarriage / number of pregnancies with evidence of an intrauterine amniotic cavity in the studied groups in %.

**Information on the regulation of the mask/test obligation in 2022 at the University Hospital (compiled by F. Hannemann):**

**Corona regulations at the University Hospital at a glance (Update Corona).**

From 26.11.2021 3G protection status was checked (fully vaccinated, tested, recovered) at central checkpoints for all patient:s and visitor:s. Individuals received a wristband with identifier. Masks (FFP2 masks) were mandatory for patients and all staff.

- 25.01.2022 Information on the definition of the status "protected" according to recommendations of the Robert Koch Institute (RKI) and Paul Ehrlich Institute (PEI). "*New scientific findings on the now predominant omicron variant of the coronavirus*"...

o 3-fold vaccination

o Convalescents with complete immunization (2 vaccinations)

o 2-fold vaccination 15- 90 days after last vaccination

o Convalescents 28-90 days after positive test.

All other patients required a daily antigen test (rapid test).

- As of 01.10.2022 FFP2 mask requirement continued, but no more testing requirement regardless of protection status for inpatients. In the Women's and Children's Hospital, testing continued to be mandatory for outpatients without 3G status.

**Supplementary Information**

**Outcome of treatments with assisted reproductive technology after SARS-CoV-2-infection**

**Violet Eckstein^1^, Katrin Glaß^1^, Marie Lessmann^1^, Jessica Schaar^1^, Anna Klimova^2^, Pauline Wimberger^1^, Maren Goeckenjan^1^**

1. Department of Gynecology and Obstetrics, University Hospital Carl-Gustav-Carus, Technische Universität Dresden, Germany
2. Institute for medical biometrics and biometry, Faculty of Medicine, Technische Universität Dresden, Germany


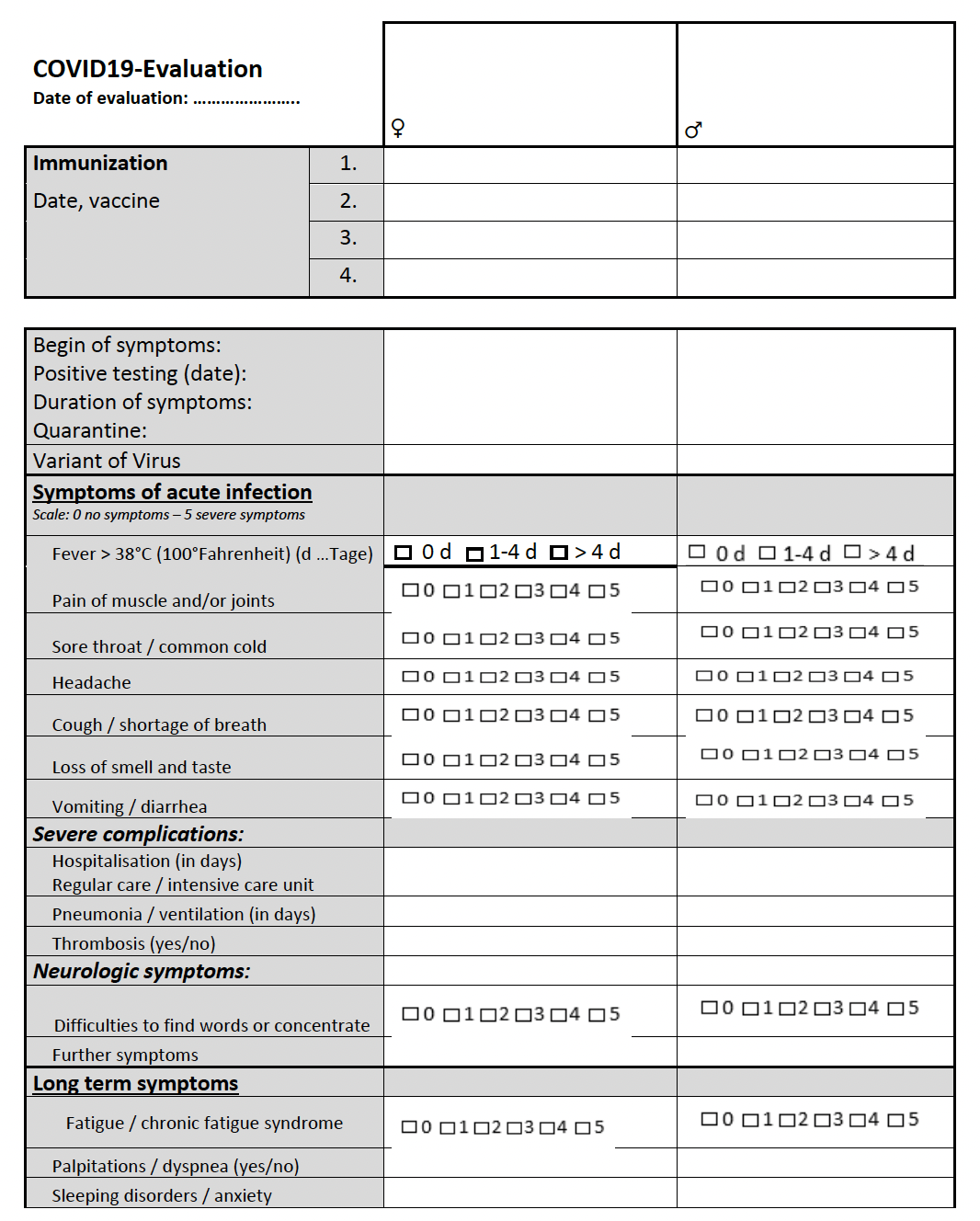

Supplement: Supplementary file 1 — Supplementary file1 (DOCX 1141 KB) [file 404_2023_7228_MOESM1_ESM.docx]
